# Creation of waterproof, TLD probes for dose measurements to validate image-based radiopharmaceutical therapy dosimetry workflow

**DOI:** 10.1088/2057-1976/accf22

**Published:** 2023-05-12

**Authors:** David P Adam, Clifford Hammer, Julia Ziege Malyshev, Wesley S Culberson, Tyler J Bradshaw, Joseph J Grudzinski, Paul M Harari, Bryan P Bednarz

**Affiliations:** 1 Department of Medical Physics, University of Wisconsin-Madison, Madison, WI, 53705, United States of America; 2 Department of Radiology, University of Wisconsin-Madison, Madison, WI, 53705, United States of America; 3 Department of Human Oncology, University of Wisconsin-Madison, Madison, WI, 53705, United States of America

**Keywords:** radiopharmaceutical therapy, dosimetry, thermoluminescent dosimeters, Monte Carlo, GEANT4

## Abstract

Voxel-level dosimetry based on nuclear medicine images offers patient-specific personalization of radiopharmaceutical therapy (RPT) treatments. Clinical evidence is emerging demonstrating improvements in treatment precision in patients when voxel-level dosimetry is used compared to MIRD. Voxel-level dosimetry requires absolute quantification of activity concentrations in the patient, but images from SPECT/CT scanners are not quantitative and require calibration using nuclear medicine phantoms. While phantom studies can validate a scanner’s ability to recover activity concentrations, these studies provide only a surrogate for the true metric of interest: absorbed doses. Measurements using thermoluminescent dosimeters (TLDs) are a versatile and accurate method of measuring absorbed dose. In this work, a TLD probe was manufactured that can fit into currently available nuclear medicine phantoms for the measurement of absorbed dose of RPT agents. Next, 748 MBq of I-131 was administered to a 16 ml hollow source sphere placed in a 6.4 L Jaszczak phantom in addition to six TLD probes, each holding 4 TLD-100 1 × 1 × 1 mm TLD-100 (LiF:Mg,Ti) microcubes. The phantom then underwent a SPECT/CT scan in accordance with a standard SPECT/CT imaging protocol for I-131. The SPECT/CT images were then input into a Monte Carlo based RPT dosimetry platform named RAPID and a three dimensional dose distribution in the phantom was estimated. Additionally, a GEANT4 benchmarking scenario (denoted ‘idealized’) was created using a stylized representation of the phantom. There was good agreement for all six probes, the differences between measurement and RAPID ranged between −5.5% and 0.9%. The difference between the measured and the idealized GEANT4 scenario was calculated and ranged from −4.3% and −20.5%. This work demonstrates good agreement between TLD measurements and RAPID. In addition, it introduces a novel TLD probe that can be easily introduced into clinical nuclear medicine workflows to provide QA of image-based dosimetry for RPT treatments.

## 1. Introduction

Dose-response relationships form the basis on which clinicians make treatment decisions in radiation oncology and substantiate the importance of dose optimization in treatment paradigms. There is evidence emerging that personalized radiopharmaceutical therapy (RPT) dosimetry can result in better outcomes due to the inter-patient variability in uptake and biological metabolism of RPT compounds (Lawhn-Heath *et al* 2022, Sgouros *et al* 2021). Personalized RPT dosimetry estimates are oftentimes image based using single-photon emission computed tomography (SPECT) or positron emission tomography (PET) scans depicting the distribution of activity within a patient as a function of time. The images are then used as input into a dose engine (e.g. dose point kernel convolution-superposition or Monte Carlo) which, integrated with pharmacokinetic modeling, provide an estimate of the three dimensional cumulative absorbed dose distribution in the patient. These approaches are collectively called voxel-level dosimetry. A variety of commercial voxel-level dosimetry software tools are currently available (Capala *et al* 2021).

Measurements serve as the basis of quality control (QC) and quality assurance (QA) in dosimetry workflows, as exhibited by the extensive methods and procedures utilized for external beam radiotherapy and brachytherapy (IAEA 2014). QA methods are much less established for image-based, voxel-level RPT dosimetry but are similarly necessary to ensure end-to-end validation of the radiation dose delivered to RPT patients. As voxel-level dosimetry becomes a clinical reality, robust QA procedures of dosimetry workflows are needed.

Thermoluminescent dosimeters (TLDs) are commonly used to accurately measure absorbed dose for a variety of radiation fields. TLDs exhibit a linear response over a large dose range that is independent of dose rate (Attix 1986). Direct measurement of the beta dose from radioisotopes using TLDs has been investigated previously but is difficult because it requires specialized techniques and TLDs with unique compositions (D’Arienzo *et al* 2020, Klein *et al* 1989). Previous investigators have looked at scoring the gamma component of I-131 using phantom measurements with good agreement between predicted and measured absorbed dose; however, these measurements required custom phantom designs or complicated experimental methods (Giap *et al* 1995, Giap *et al* 1995, Marcatili *et al* 2013).

The rapid introduction of RPT into routine clinical practice calls for integratable QA solutions for end-to-end dosimetric validation of voxel-level RPT dosimetry workflows. In this work we manufactured watertight probes to house TLDs in a commercial Jazczcak phantom for the measurement of absorbed dose from I-131. The probe was designed with three primary criteria: (i) ease of manufacturing, (ii) ease of integration with commercially available nuclear medicine phantoms, and (iii) accuracy in line with the current state of literature. After the probe was designed and manufactured, it was implemented in a Jaszczak phantom study. In this study, the absorbed dose measured by TLDs was compared against a RAPID, an image-based RPT dosimetry workflow utilizing images acquired with a SPECT/CT scanner. The results are presented herein and provide confidence in our image based dosimetry methods such that they can be readily applied for patient cases.

## 2. Materials and methods

### 2.1. Creation of probe

**Figure 1.**
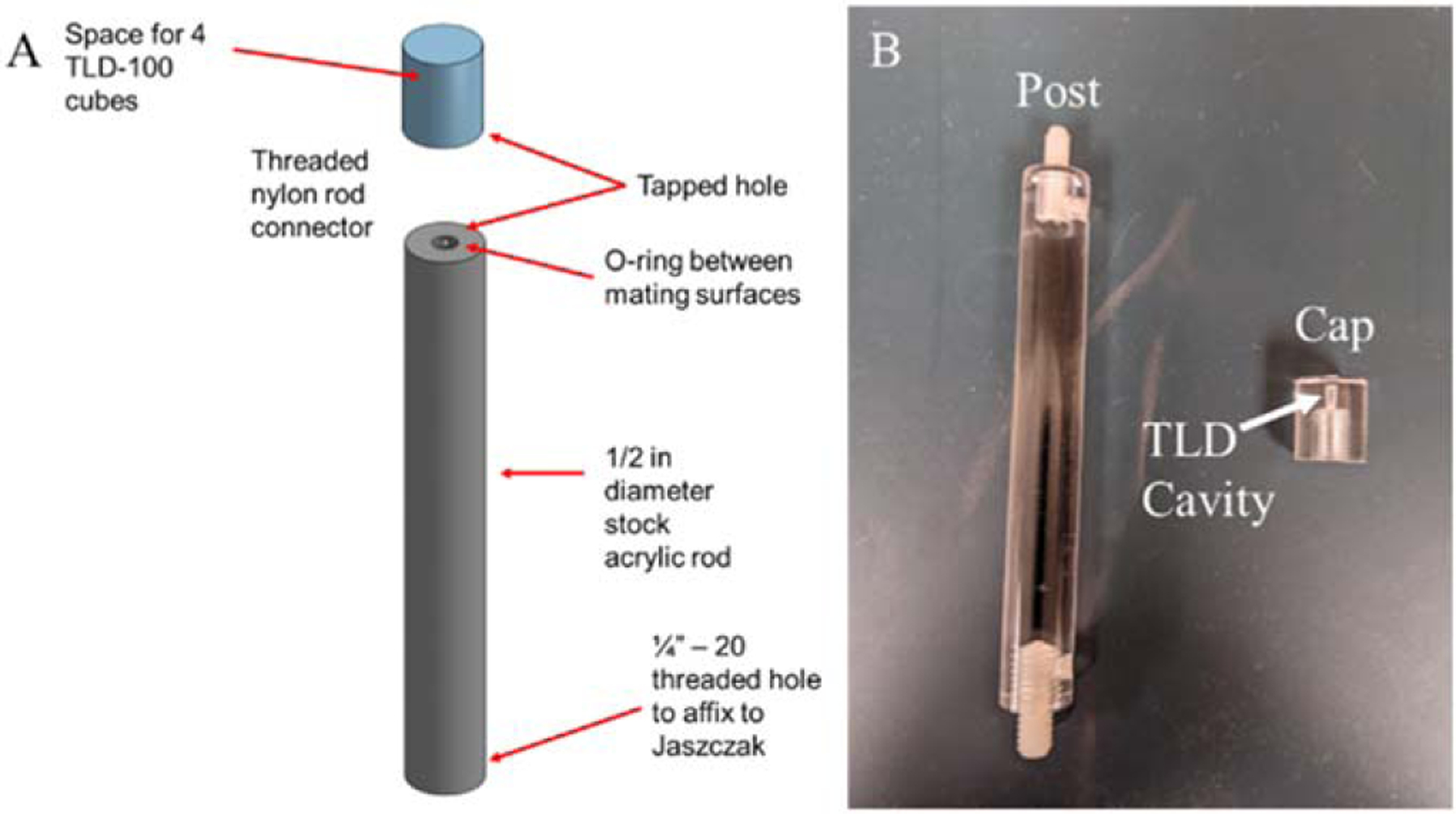
(a) Graphical depiction and (b) photograph of the probe assembly.

The TLD probe assembly is detailed in figure 1. It consists of two parts, a post and a cap, both of which are machined from a standard 1/2 inch (12.7 mm) diameter acrylic rod. The post is 9.65 cm tall and has two tapped holes at each end in which threaded nylon studs are inserted. One nylon stud is used to affix the probe assembly to a standard ¼’−20 threaded hole, commonly found on commercially available nuclear medicine phantoms and the other stud is used for attaching the cap onto the post assembly. The cap is 1.52 cm tall and has two machined holes in it. One of the holes is tapped to screw onto the threaded nylon stud and attach the cap to the post. The second machined hole is a 4.3 mm tall and 1.414 mm diameter cavity at the end of the tapped hole; it is used to hold four 1 mm × 1 mm × 1 mm TLD-100 (LiF:Mg,Ti) microcubes stacked atop each other. An o-ring gasket is affixed between the rod and the cap to provide a watertight seal so that the TLDs remain dry.

### 2.2. Experimental setup

**Figure 2.**
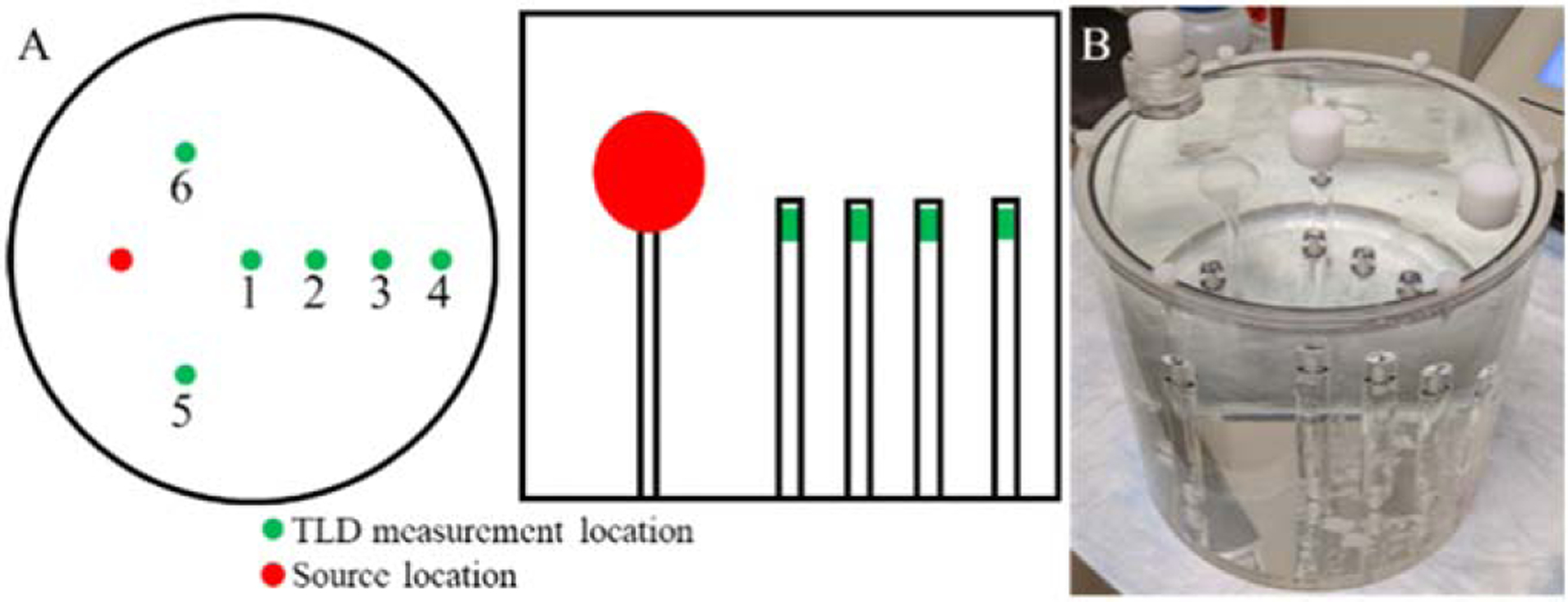
(a) Schematic showing axial and coronal views and (b) photograph of the phantom setup.

Six probes were manufactured for the experiment to measure the dose at multiple points in a 6.4 L Jaszczak phantom. The experimental setup is depicted schematically figure 2(a) and a photograph is depicted in figure 2(b). The source was a 16 ml hollow sphere in which I-131 was placed within; the remainder of the phantom was non-radioactive. Six probes were placed at different points in the phantom. Four TLD-100 microcubes were placed into each probe.

#### 2.2.1. Idealized GEANT4 simulations

Idealized GEANT4 simulations (denoted ‘Ideal’) were performed to both estimate an appropriate amount of activity to administer such that a sufficient absorbed dose would be imparted to the TLDs to be within a linear portion of their dose-response and to be a benchmark. GEANT4 10.07.p02 was used and the geometry replicating the experimental setup was created using constructed solid geometry solids. G4GeneralParticleSource was used to create a spherical source of I-131. Additionally, absorbed dose was scored using a primitive scorer of G4MultiFunctional-Detector in a 5 mm × 5 mm × 5 mm cube near the top of each probe. The physics list utilized was ‘emlivermore.’ The materials utilized were predefined materials in GEANT4 including ‘G4_AIR,’ ‘G4_WATER,’ and ‘G4_POLYSTYRENE.’ The results from the simulation were in units of Gy per initial particle history (*D*_*history*_) and the absorbed dose (*D*) was calculated according to,

(1) $$D=\tilde{\text{A}}xD_{\text{history}}$$

where,

(2) $$\tilde{\text{A}}\text{=}\frac{A_{\text{admin}}}{\lambda }(1-\text{exp}(\lambda t_{\text{exposed}}))$$

Note, *A*_*admin*_ is the initial administered activity, λ is the I-131 decay constant, and *t*_*exposed*_ is the time of exposure.

The amount of administered activity was iterated until each of the TLD probes was estimated to conservatively receive an absorbed dose in the linear dose-response region of the TLD-100 microcubes. The simulation concluded that approximately 20 mCi of I-131 activity would be necessary to elicit the range of desired absorbed doses to the TLDs. Table 1 depicts the parameters used for the experimental setup and includes the actual amount of administered activity and time of exposure during the experiment.

In addition to scoring dose, the photon energy spectrum was scored at each dose measurement volume in order to provide an energy correction against the polyenergetic beam used for the TLD calibration. The mean energy of the spectrum of photons was calculated for each probe location by tallying the energy of the volumetric flux.

#### 2.2.2. TLD measurement and corrections

The TLDs used in this work were TLD-100 microcubes (LiF:Mg,Ti) (Thermo Fisher Scientific, Waltham, MA) with nominal dimensions of 1 mm × 1 mm × 1 mm. The TLDs were annealed using the standard University of Wisconsin-Madison Medical Radiation Research Center (UWMRRC) annealing protocol(Nunn *et al* 2008). Prior to each irradiation, TLDs were placed in an aluminum tray and annealed at 400 °C for 1 h, allowed to cool to room temperature, and then annealed at 80 °C for 24 h. There was a 24 h waiting period between the completion of the 80 °C anneal and irradiation. TLDs were read with a Harshaw (Oakwood Village, OH) 5500 hot gas reader 24 h after irradiation, and the same annealing procedure was performed prior to the next set of measurements. TLDs from a given set of measurements were read out at the same time to avoid the effect of any day-to-day variability of the TLD reader. TLDs were read by preheating to 100 °C, and then collecting signal as the temperature was increased to 350 °C at a rate of 15 °C s^−1^. The temperature was then held at 350 °C for a total collection time of 26.7 s. There was no fading correction applied as the glow peaks of interest were sufficiently stable over the time interval of this experiment.

TLDs exhibit significant energy dependence at energies below 1 MeV. Nunn *et al* found that TLD-100 dosimeters had up to a 40% overresponse relative to Co-60 at x-ray energies (Nunn *et al* 2008). Matching experimental and calibration beam qualities is ideal but often not possible. For this work, the UWMRRC’s NIST-traceable UW-250M beam was determined to have the closest in-depth mean energy (111 keV) relative to the experimental mean energy range. Calibration TLDs were given multiple NIST-traceable absorbed doses covering the expected experimental dose range. Additional energy corrections were applied to account for the difference in energies by interpolating between the UW-250M and Cs-137, the two closest NIST-traceable mean-energy matches to I-131 available at the UWMRRC at the time of the experiment.

### 2.3. SPECT/CT image acquisition and analysis

The image acquisition parameters that were used are described in detail elsewhere (Adam *et al* 2022). SPECT/CT acquisitions were conducted using a GE Optima NM/CT 640 with a high energy general purpose collimator. The images were acquired at 7.1 days after the activity was administered to reduce the potential impact of scanner dead time. The acquisition and reconstruction parameters were the same as the quantitative calibration scan. All images were acquired over 360 degrees into 128 × 128 pixel matrices per angle with 120 projections and 30 s per projection. Body contouring was enabled. A photopeak energy window centered at 364 keV and 20% in width and a triple energy window scatter correction 20% in width. A CT was acquired after the SPECT acquisition for both CT-based attenuation corrections and to provide material density for absorbed dose calculations.

The images were reconstructed using the GE Xeleris 4.0 ordered subset expectation maximum (OSEM) algorithm with 10 iterations and 10 subsets with CT-based attenuation correction and with GE’s collimator detector response modeling. No post-reconstruction filtering was applied. The reconstructed SPECT image matrices were 128 × 128 × 128 voxels with a voxel size of 4.42 × 4.42 × 4.42 mm^3^ and the reconstructed CT images had a voxel size of 0.98 × 0.98 × 5.0 mm^3^.

### 2.4. RAPID—An image-based dosimetry workflow

An in-house radiopharmaceutical dosimetry platform called RAPID was used to estimate the absorbed dose distribution in the phantom and TLD probes from the recorded SPECT/CT images of the phamtom (Besemer *et al* 2018). The input to RAPID was the recorded SPECT/CT image of the phantom and TLD probe arrangement. The acquired SPECT images define the radionuclide activity in each voxel and the absorbed dose rate distribution is calculated on the corresponding CT which defines the material composition and mass density of the simulation geometry based upon the conversion defined by Schneider (Schneider *et al* 2000). The dose computation in RAPID is Monte Carlo based utilizing GEANT4 9.6.p02 and the physics list utilized in this investigation is based upon ‘emstandard.’ For further details, refer to (Besemer *et al* 2018).

The SPECT images, which are recorded in counts, were converted into units of absolute activity concentration in order to be used in the dose estimation. A calibration factor was previously obtained from a 5.64 L cylindrical phantom scan in the same scanner. This calibration factor was applied to the reconstructed SPECT image to convert the recorded SPECT image data into units of activity. All pixels outside of the 16 ml sphere were set to zero to prevent background noise from impacting dose estimation. Next, a previously measured recovery coefficient for the 16 ml hollow sphere was applied to the source in order to correct the partial volume effects and recover the activity which appears to spill out of the sphere due to limitations of the imaging system (Adam *et al* 2022).

The corrected, quantified SPECT/CT data was used to estimate a voxel-level dose rate distribution in the phantom. The dose rate in each voxel was then integrated assuming single timepoint exponential decay to produce a total absorbed dose distribution in the phantom.

The SPECT data was interpolated to the same resolution as the CT using the 3DSlicer ‘Resample image (BRAINS)’ module. The dose calculation grid was of dimensions 247 × 253 × 52 voxels with a 0.977 × 0.977 × 5.00 mm^3^ voxel size. The simulation was run with 1E6 decays per source voxel. Simulations were run on the UW Center for High Throughput Computing (CHTC). 3D Slicer 4.11.20210226 was used for the segmentation and analysis to compute the mean absorbed dose to each probe volume (Pieper *et al* 2004, Pinter *et al* 2012).

## 3. Results

**Figure 3.**
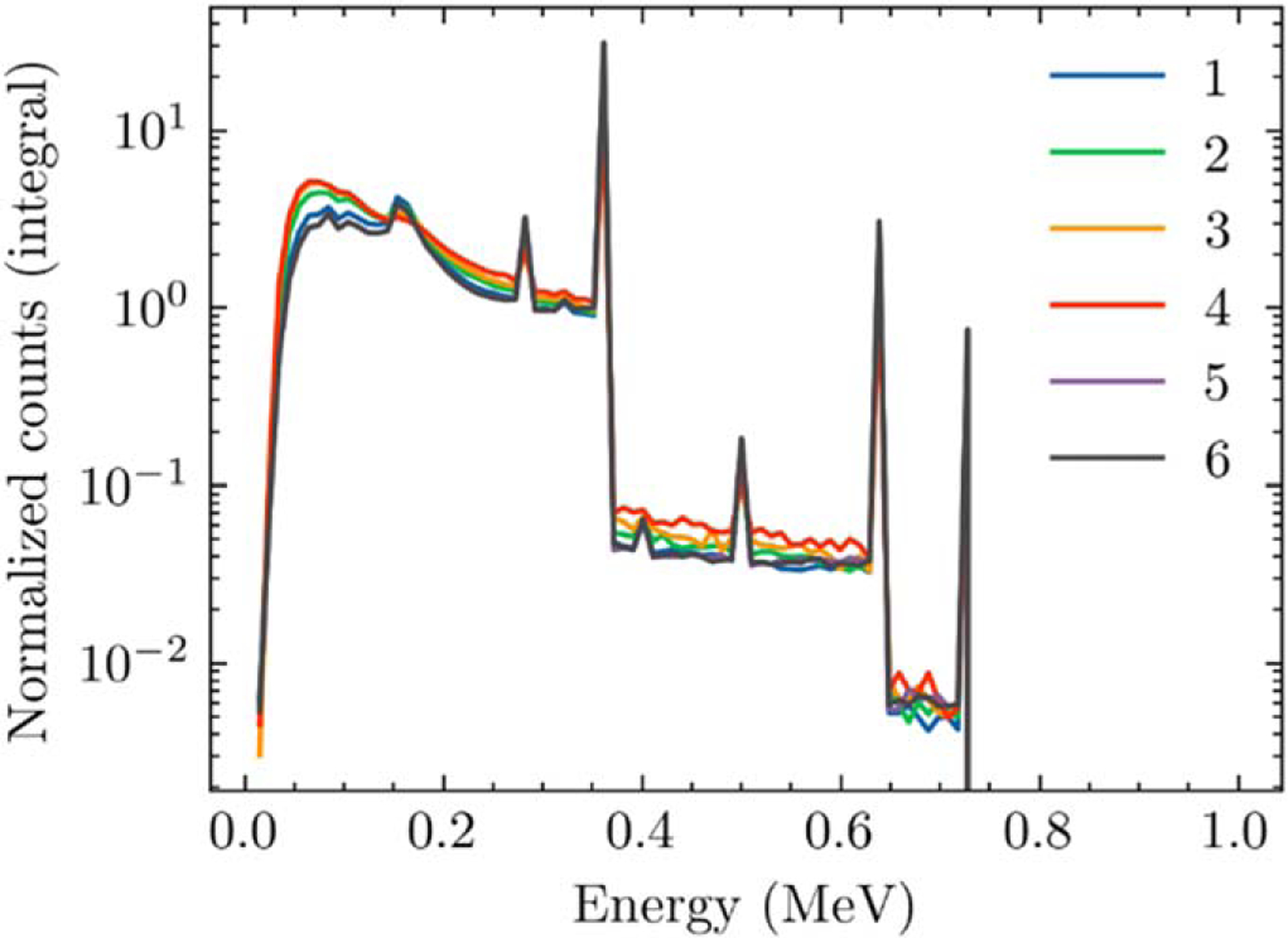
Tallied, normalized volumetric energy flux at each of the probe locations. These results were utilized for energy corrections to the TLD measurements.

The results of the energy spectra scored for each of the probe locations is presented in figure 3. The integral of each curve is normalized to unity. Recall these GEANT4 simulations were performed to identify the NIST traceable calibration beam quality and to provide an energy correction against the polyenergetic beam used for the TLD calibration. The results are consistent with decay scheme for I-131, evidenced primarily by the photopeak at 364 keV.

The mean energy of each spectra for each probe location (1–6) was 234.3, 208.0, 194.8, 191.8, 249.7, and 249.7 keV, respectively. As outlined in the methods section, these mean energies were higher than but closest to the UW-250M beam, with Cesium-137 being the next established beam at energies above. This provided motivation to use the UW-250M beam quality with interpolated energy corrections for calibration.

**Figure 4.**
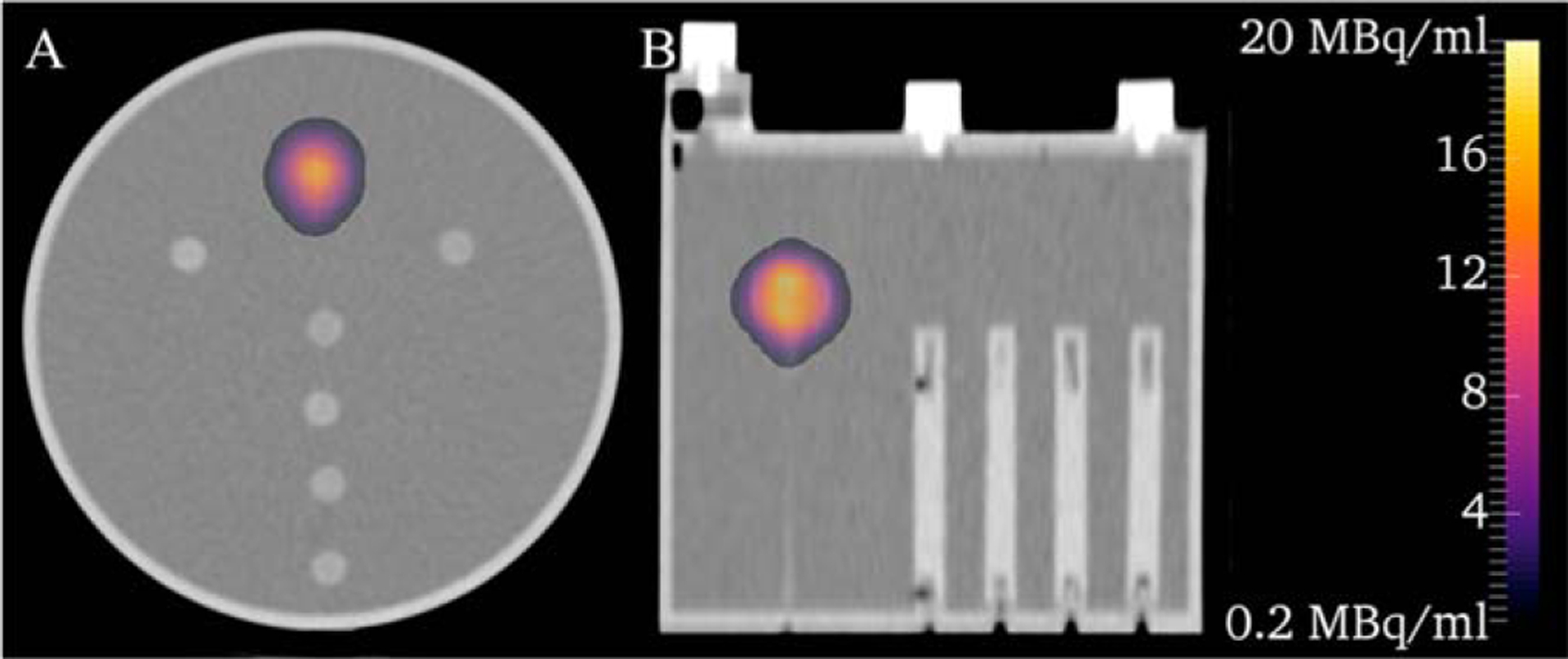
Activity distribution colorwash without PVC superimposed over a low dose CT of the phantom.

Figure 4 depicts the SPECT/CT images where a colorwash of the activity concentration is shown superimposed over the low dose CT of the phantom. Partial volume corrections (PVC) are not present in the presented images because the PVC methods utilized are volume averaged and cannot be applied at the voxel level. Prior to the application of a recovery coefficient, the mean activity concentration of the source sphere was 12.8 MBq ml^−1^ (−49.5% compared to administered); after the application of the recovery coefficient, it was 23.1 MBq ml^−1^ (−9.0% compared to administered). Some Gibbs ringing artifacts are present and likely attributable to the use of GE’s collimator detector response modeling (Ljungberg & Sjögreen-Gleisner, 2011) but could also be related to energy subtraction during scatter correction. After resampling, the mean activity concentration in the source sphere 5.2% smaller than the original SPECT image.

**Figure 5.**
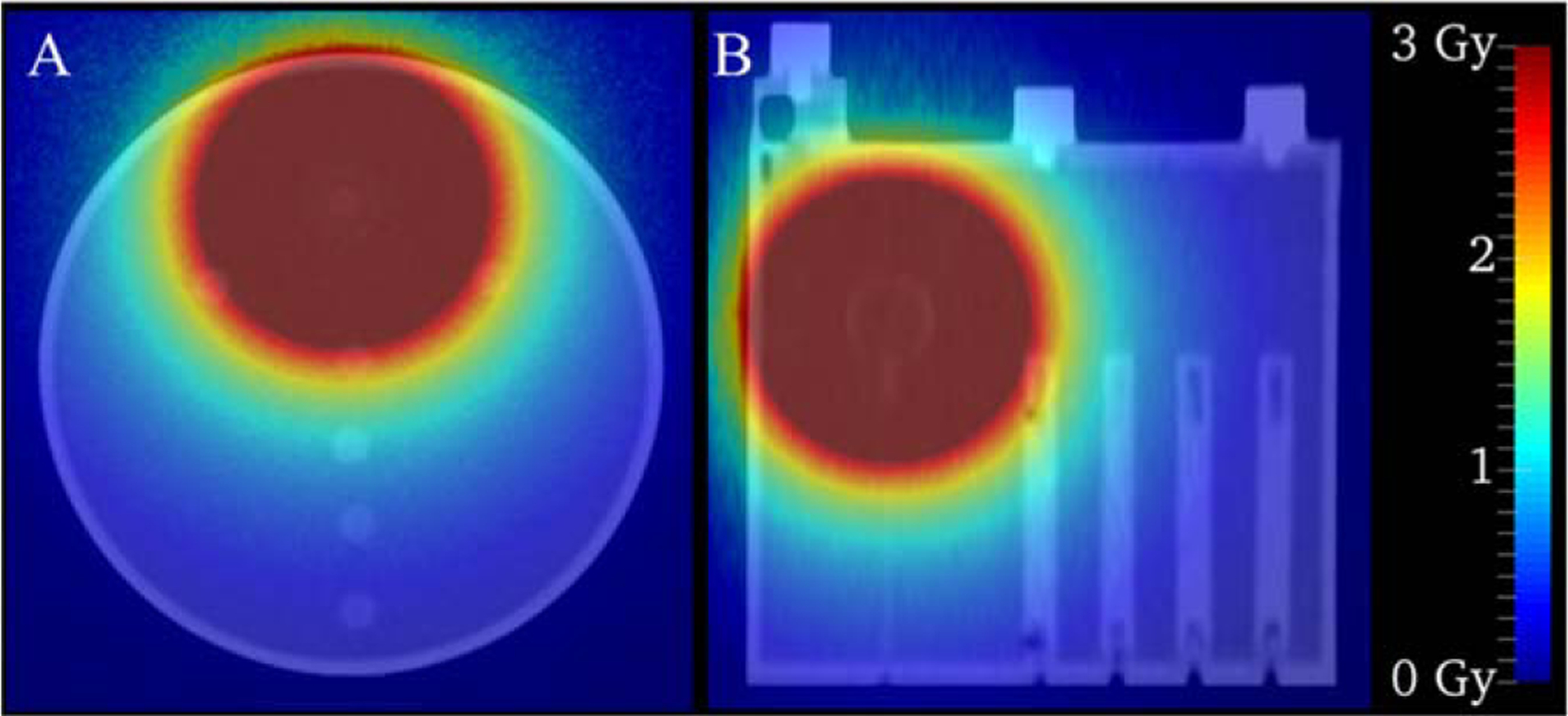
Dose distribution estimated in the phantom/probe arrangement. The colorscheme has been thresholded from 0 to 3 Gy to more clearly show differences in dose deposition near the TLD probe locations.

Figure 5 shows the dose distribution colorwash calculated by RAPID superimposed over the low dose CT. The colorwash has been thresholded from 0–3 Gy, otherwise the colorscheme would overwhelmingly manifest the dose in the source region and not show any discernable differences in the region of the phantom where the TLD probes are located.

Table 2 contains a comparison of the absorbed dose esimated using the RAPID, the idealized GEANT4 simulation, and the TLD measured dose for all six probe locations including the standard deviation of the four TLDs per probe location. The statistical uncertainty was kept under 3.8% and 2.3% for RAPID and the idealized case, respectively.

The differences between the measured TLD absorbed dose and the RAPID ranged from −5.5% to +0.9% difference in which the TLD dose was used as the reference. In addition, the difference between the measured and the ideal scenario was calculated and ranged from −4.3% and −20.5%, attributable to slight differences between the idealized and image-based scenario. The standard deviation in the TLD measurements for each probe ranged from 1.0% to 10.3%. Two probes (1 and 5) had TLD outliers which were kept in the analysis and were defined as measurements exceeding 6% of the mean of the group (e.g. two standard deviations of the expected repeatability of 3%). A very conservative uncertainty of 10% at k = 2 confidence level was assumed for the TLD results. This is primarily due to the interpolation from the UW-250M beam to the various measurement-point energy responses (beam-matched TLD measurements of this sort would have expected uncertanties of ~5% at the UWMRRC). All differences between the measurements and image-based dosimetry were nearly within the 10% expected uncertainty.

## 4. Discussion

Good agreement was shown between the SPECT/CT image-based workflow and the measured TLD values; the largest difference was a 5.5% overestimation using the image-based workflow. In line with previous work (Adam *et al* 2022), the necessity of PVC is required for accurate measurements of activity and subsequently the calculated absorbed dose distribution. Without PVC, the activity in the sphere was underestimated by 49.5% in the SPECT images. The idealized GEANT4 scenario used to calculate the amount of activity predicted doses greater than the measured by up to 20.5%. This disagreement is likely due to key differences in experimental and Monte Carlo geometries, including differences in resolution, and differences between the idealized scoring volume compared to TLD dimensions. The resolution of the SPECT images are much coarser than the perfect sphere assumed for the idealized scenario and some Gibbs ringing artifacts were observed on the SPECT image. Scoring volumes were similarly slightly different. Resampling could account for some differences as well. All differences were within calculated uncertainties in the activity concentration (23.6% uncertainty) for a 16 ml sphere. (Adam *et al* 2022). These uncertainties are of similar magnitude to those reported by Gregory *et al*, where they reported an uncertainty in the activity determination to be 16% for a 5 cm sphere (Gregory *et al* 2019).

The experimental setup had a few limitations compared to typical nuclear medicine phantom studies. First, the amount of activity administered to the sphere was a high activity concentration, larger than what would likely be utilized in routine scanner QA/QC. The amount of administered activity was explicitly calculated in order to elicit a linear response out of the TLD probes placed throughout the phantom. To reduce the amount of administered activity, different spatial configurations of the probes could be utilized. This could easily be accomplished during typical Jaszczak PVC imaging studies; this study utilized one source to minimize confounding factors which may increase uncertainties. Second, activity was only administered to a single, isolated source region; no activity was administered to the phantom outside of the source cavity. This was done to minimize the possibility of radioactive contamination and avoid the necessity of sequestering the probes to decay to background levels. Since the probe is watertight, will tolerate radioactive contamination, and will protect the TLDs, the end user must decide how long they can wait to read out the TLDs. In the case of I-131, this would have required approximately 80 days of waiting if they were exposed to contamination. Third, the probes should be subject to tests assessing the reproducibility of the measurements. Confidence was imparted to our results for this work by utilizing 4 separate TLDs in each probe and it is our hypothesis that the inter-study reproducibility should emerge through subsequent use of the probes in future research and clinical work.’

The absorbed dose of the low dose CT scan from the SPECT/CT imaging protocol was not accounted for in this study because it was expected to have a negligible impact on the measured absorbed dose. Care must be taken if the expected measured absorbed dose is low enough such that the dose from the CT scan cannot be ignored.

The assumed uncertainty of 10% were due to multiple factors. The uncertainty in sorting, repeatability, and readout of the TLDs were calculated to be 3.6% in summary; by including an additional amount for the absorbed-dose calibration, this factor was increased to 4.4%. Another 0.6% was added when accounting for the uncertainty in applying known energy correction factors and another 1%–2% was added which is due to the uncertainty of the known energy interpolation. The interpolation was conducted in a relatively flat portion of the energy response curve; however, this portion of the energy-response curve has the least amount of data from which to interpolate upon. Lastly, an additional 3%–4% uncertainty were very conservatively assumed to be attributable to overall setup and modeling uncertainties, rendering the assumed final uncertainty of 10%.

## 5. Conclusion

A waterproof, TLD holding probe was created and utilized to measure the dose from I-131 in a nuclear medicine phantom and compared against an image-based RPT dosimetry workflow. The TLDs showed good agreement to the estimated absorbed dose and provided confidence that dosimetry estimates performed using Monte Carlo-based methods can be confidently applied to patient cases. Recognizing the increasing usage of radiopharmaceuticals in clinical practice and the necessity of developing techniques and methods for QA/QC of RPT dosimetry estimates, the probe described in this document offers an accurate and easily implementable tool in current nuclear medicine QA/QC workflows for end-to-end dosimetry validation.

## Figures and Tables

**Table 1.** Parameters used for absorbed dose calculation to TLD probe locations.

Administered activity	748 MBq
T_1/2_	8.0197 days
*λ*	0.08643 days^−1^
Days to decay	16.87 days
Integrated activity	5.737E + 14 Bq·s
Primary histories	2E + 09
Volume of Source Sphere	16 ml
Activity concentration	46.7 MBq/ml

**Table 2.** Measured versus image based absorbed dose and percent differences for six probe locations.

Probe	Measured (Gy)	Measured Standard Deviation (%)	RAPID (Gy)	Ideal (Gy)	Percent Difference (Measured - RAPID)	Percent Difference (Measured - Ideal)
1	2.37	10.3	2.36	2.51	0.71	−5.92
2	0.98	3.4	0.99	1.07	−1.13	−9.02
3	0.49	1.9	0.51	0.55	−4.17	−11.18
4	0.27	1.0	0.27	0.29	−3.58	−10.84
5	2.06	7.3	2.17	2.48	−5.48	−20.52
6	2.49	2.4	2.47	2.60	0.95	−4.30

## Data Availability

The data cannot be made publicly available upon publication because they are not available in a format that is sufficiently accessible or reusable by other researchers. The data that support the findings of this study are available upon reasonable request from the authors.
